# Mechanisms Contributing to Differential Regulation of *PAX3* Downstream Target Genes in Normal Human Epidermal Melanocytes versus Melanoma Cells

**DOI:** 10.1371/journal.pone.0124154

**Published:** 2015-04-16

**Authors:** Danielle Bartlett, Glen M. Boyle, Mel Ziman, Sandra Medic

**Affiliations:** 1 School of Medical Sciences, Edith Cowan University, Perth, Australia; 2 Cancer Drug Mechanisms Group, Division of Cancer & Cell Biology, QIMR Berghofer Medical Research Institute, Brisbane, QLD, Australia; 3 School of Pathology and Laboratory Medicine, University of Western Australia, Perth, Australia; 4 Curtin Health Innovation Research Institute of Ageing and Chronic Disease, Curtin University, Perth, Australia; University of Connecticut Health Center, UNITED STATES

## Abstract

Melanoma is a highly aggressive and drug resistant form of skin cancer. It arises from melanocytes, the pigment producing cells of the skin. The formation of these melanocytes is driven by the transcription factor *PAX3* early during embryonic development. As a result of alternative splicing, the *PAX3* gene gives rise to eight different transcripts which encode isoforms that have different structures and activate different downstream target genes involved in pathways of cell proliferation, migration, differentiation and survival. Furthermore, post-translational modifications have also been shown to alter the functions of PAX3. We previously identified PAX3 downstream target genes in melanocytes and melanoma cells. Here we assessed the effects of *PAX3* down-regulation on this panel of target genes in primary melanocytes versus melanoma cells. We show that PAX3 differentially regulates various downstream target genes involved in cell proliferation in melanoma cells compared to melanocytes. To determine mechanisms behind this differential downstream target gene regulation, we performed immunoprecipitation to assess post-translational modifications of the PAX3 protein as well as RNAseq to determine *PAX3* transcript expression profiles in melanocytes compared to melanoma cells. Although PAX3 was found to be post-translationally modified, there was no qualitative difference in phosphorylation and ubiquitination between melanocytes and melanoma cells, while acetylation of PAX3 was reduced in melanoma cells. Additionally, there were differences in *PAX3* transcript expression profiles between melanocytes and melanoma cells. In particular the *PAX3E* transcript, responsible for reducing melanocyte proliferation and increasing apoptosis, was found to be down-regulated in melanoma cells compared to melanocytes. These results suggest that alternate transcript expression profiles activate different downstream target genes leading to the melanoma phenotype.

## Introduction

Melanoma is the most aggressive form of skin cancer with the annual incidence consistently increasing worldwide [1]. The 5-year survival rate for early stage melanoma patients is high (98–95%), while for advanced stage patients this is reduced to less than 50% [2]. With limited treatment options for advanced stage patients and new therapies showing success in only a subset of patients [3], it remains important to better understand mechanisms driving melanoma development and progression. Identifying differences in key regulators of cellular processes in normal skin melanocytes and melanoma cells may provide strategic clues to the process of melanomagenesis and targets for therapy.

Melanomas arise from melanocyte cells of the skin. The transcription factor PAX3 is at the top of the hierarchy of genes that regulate melanocyte specification, differentiation, proliferation, survival and migration during embryonic development [4,5]. PAX3 is also highly expressed in melanoma, where it has been shown to contribute to cell survival, differentiation, migration and proliferation [6–9].

We have previously shown persistent PAX3 expression in developing melanoblasts [10] and in melanocytes of normal adult skin [7]. We have also identified PAX3 expression at all stages of melanoma progression [7]. Our analysis of PAX3 downstream targets in melanocytes and melanoma cells showed that while a subset of target genes are similarly regulated by PAX3 in melanoma and melanocyte cells, particularly those that regulate maintenance of an undifferentiated ‘stem cell’ phenotype, PAX3 differentially regulates target genes that are associated with cell proliferation and survival in melanoma cells relative to melanocytes [8]. Since this differential regulation of melanoma cells by PAX3 may play a role in melanomagenesis, we sought to investigate the possible mechanisms behind this differential target gene selection.

One such mechanism may be found in expression profiles of alternate *PAX3* transcripts (*PAX3A-H*), as these have previously been shown to differentially affect cell proliferation, migration and survival through differential activation of downstream target genes [11,12]. Furthermore, post-translational modifications of the PAX3 protein, namely acetylation, phosphorylation and ubiquitination, have been shown to affect neural and skeletal muscle precursor cell differentiation and stem-cell maintenance [13–19]. It is not known to what extent alternate transcript expression and post-translational modifications affect differential regulation of downstream target genes in cells of the melanocytic lineage and therefore their role in melanoma development and progression.

Here we first confirmed the differential PAX3 mediated regulation of downstream target genes, and then compared the *PAX3* transcript expression profiles and post-translational modifications of PAX3 in several melanoma cell lines compared to normal melanocytes *in vitro*. While we saw no striking differences in post-translational modifications of PAX3 isoforms, we did observe differences in alternate *PAX3* transcript expression profiles and in translation of mRNA between melanocytes and melanoma cells. The difference in *PAX3* transcript expression profiles could, if proven in a larger cohort of cell lines and melanoma tissue samples, provide a tool for stratification of melanomas for diagnosis and treatment.

## Methods

### Cell culture

Human melanoma and melanocyte cell cultures were maintained as a monolayer at 37°C, in 5% CO_2_. Primary cultures of adult human epidermal melanocytes (NHEM-a (P), PromoCell) [20] and neonatal human epidermal melanocytes (NHEM-n, PromoCell) [21] were maintained in Melanocyte Growth Media (PromoCell), whereas adult melanocyte primary culture (NHEM-a (I), Gibco) [22] was maintained in 254 media (Gibco), supplemented with HMGS-2 (Gibco). Metastatic melanoma cell lines (A2058 [23], M14 [24], SKMEL2 [25], SKMEL5 [25] and UACC62 [26]) were cultured in Dulbecco’s modified Eagle’s medium (DMEM). Primary melanoma cell lines MM200 [27], MM329 [28], MM540 [29] and MM622 [30] and metastatic melanoma cell line MM229 [31] were maintained in RPMI-1640 media whereas WM115 was cultured in Eagle’s minimum essentials media (EMEM). All media was supplemented with 10% foetal bovine serum (FBS) (Sigma Aldrich), L-glutamine (300 μg/ml), penicillin (400 U/ml) and streptomycin (50 μg/ml).

### *PAX3* Silencing

Silencing of all *PAX3* transcripts was accomplished using pre-designed *PAX3*-specific Silencer Select siRNA (s10059 and s224172, Ambion), and Lipofectamine RNAiMAX Transfection Reagent (Invitrogen) following the manufacturer’s recommendations. Briefly, 1×10^5^ melanoma cells or 2×10^5^ melanocytes were seeded in a six-well plate 24 hours prior to transfection with 10 nM of *PAX3*-specific siRNA (s10059 and s224172 individually, or in combination), or negative control siRNA (Negative Control #1 siRNA, Ambion) using Lipofectamine RNAiMAX Transfection Reagent (Invitrogen). Each silencing experiment also included a positive control, transfection with Silence Select GAPDH siRNA (Ambion), as well as non-treated cells. Efficient *PAX3* knock-down was confirmed after 24, 48 and 72 hours post-transfection by RT-qPCR and western blot. The changes in the expression of PAX3 targets, following *PAX3* knock-down, were then analysed by RT-qPCR and quantified by calculating the fold change (ΔΔCt) relative to 18S and to the negative control. The percentage of expression of each of the downstream target genes was compared to the percentage of remaining *PAX3* expression, following *PAX3* silencing, using a student’s T-test.

### RT-qPCR

Total RNA was isolated using the Isolate RNA mini-kit (Bioline) and RNA quality was confirmed on the Bioanalyser (Agilent), before 500 ng of total RNA (or 200 ng for silencing experiments) was reverse transcribed using the Omniscript RT kit (Qiagen), according to the manufacturer’s instructions. qPCR was performed on iQ5 (BioRad) using KAPA SYBR FAST qPCR Master Mix (KapaBiosystems). Primers for total *PAX3* mRNA are detailed in S1 Table. Gene expression levels were compared between melanocyte cells and primary or metastatic melanoma cell lines using the Kruskal-Wallis test and additional between-group comparisons were performed using the Mann-Whitney U test.

### RNA sequencing

Library construction and 100 bp paired-end sequencing were performed by the Australian Genome Research Facility (AGRF) Services on Illumina HiSeq2000 systems using 1–10 μg of total RNA from each sample (neonatal and adult primary melanocytes and primary MM540 and metastatic M14 cell lines). Data alignment to known *PAX3* sequences was analysed for alternative transcript identification. The sequence reads generated were cleaned and aligned against the human genome using the Tophat aligner. Raw gene counts that were mapped to known gene sequences in the human genome were used to calculate differential gene expression in each sample. The Cufflinks tool was used to assemble the transcripts and to check for novel transcript expression. Cuffmerge and Cuffdiff were then used to first merge the compared samples and then analyse differential expression. Expression levels were then calculated as number of reads per kilobase of exon model per million mapped fragments.

### Immunoprecipitation

PAX3 proteins were immunoprecipitated from the total nuclear protein fraction with either rabbit polyclonal (Abcam, 2.5μg/ml), or rabbit monoclonal (Life Technologies, clone 16H22L10) antibodies. The antibody/antigen complex was immunoprecipitated using anti-IgG agarose beads (Rockland), or Pierce Direct IP Kit (Pierce Biotechnology). Both direct and indirect methods of immunoprecipitation were performed to optimise the results. Precipitated proteins were run on a NuPAGE Bis-Tris precast gel. The following antibodies were used for western blotting: anti-PAX3 (mouse monoclonal, DSHB, 1/1000; or rabbit monoclonal, Life Technologies, clone 16H22L10), anti-phosphoserine (rabbit polyclonal, Abcam, 2.5μg/ml), anti-acetyl-lysine (rabbit polyclonal, Abcam, 2.5μg/ml), and anti-ubiquitin (rabbit polyclonal, Abcam, 2.5μg/ml). When immunoprecipitation was performed using anti-phosphoserine, anti-acetyl-lysine or anti-ubiquitin antibodies, membranes were probed with anti-PAX3 (mouse monoclonal, DSHB, 1/1000).

### Western blot

Neonatal and adult melanocyte cells and primary and metastatic melanoma cells were lysed using cell lysis buffer (50 mM pipes, 85 mM KCl, 1% Nonidet P-40, protease inhibitor cocktail (PIC, Roche), pH 8.0). Following centrifugation the supernatant (containing cytosolic proteins) was removed and nuclear proteins were extracted with nuclear lysis buffer (50 mM Tris, 10 mM EDTA, 1% SDS, 1× PIC, pH 8.0). 20 μg Total nuclear protein (20 μg) was loaded onto NuPAGE 4–12% Bis-Tris gels (Life Technologies) and separated by SDS-PAGE. Proteins were transferred onto nitrocellulose membranes (Bio-Rad), probed with mouse monoclonal PAX3 antibody (DSHB, 1/1000) and visualised using the Westernbreeze chemiluminescent detection kit (Life Technologies) as per the manufacturer’s instructions. To quantify PAX3 protein levels, membranes were stripped using Restore Western Blot Stripping Buffer (Thermo Scientific) and re-probed with anti-actin antibody (rabbit polyclonal, Abcam, 1/1000). The levels of each protein were assessed by densitometry using the GS-800 Calibrated Densitometer (Bio-Rad). PAX3 protein levels were compared between melanocyte cells and primary or metastatic melanoma cell lines using a one-way ANOVA.

### Immunocytochemistry

5×10^4^ cells per cover slip were grown for 24 hours. Cells were fixed in 4% paraformaldehyde and permeabilised for 15 minutes at room temperature with PBS containing 0.2% Triton-X100 (0.2% PBST), then blocked with 10% normal goat serum (NGS) in 0.2% PBST for 1 hour. Cells were stained overnight at 4°C with mouse monoclonal PAX3 antibody (DSHB) at 1/50 dilution. This was followed by incubation with anti-mouse IgG conjugated with Dylight-550 for 1 hour. All antibodies were diluted in 0.2% PBST containing 1% NGS. Cover slips were mounted onto microscope slides with Prolong Gold Antifade Reagent with DAPI (Invitrogen). Slides were viewed with an Olympus BX41 epi-fluorescent microscope.

## Results

### Differential regulation of PAX3 downstream targets in melanocytes and melanoma cells

We previously identified PAX3 binding to specific downstream target genes associated with melanocyte differentiation, proliferation, migration and cell survival and showed their apparent differential regulation by PAX3 in melanocytes and melanoma cells [8]. To further confirm differential target gene regulation we silenced *PAX3* in several primary melanocytes and melanoma cell lines, and analysed changes in the target gene expression.

**Fig 1:**
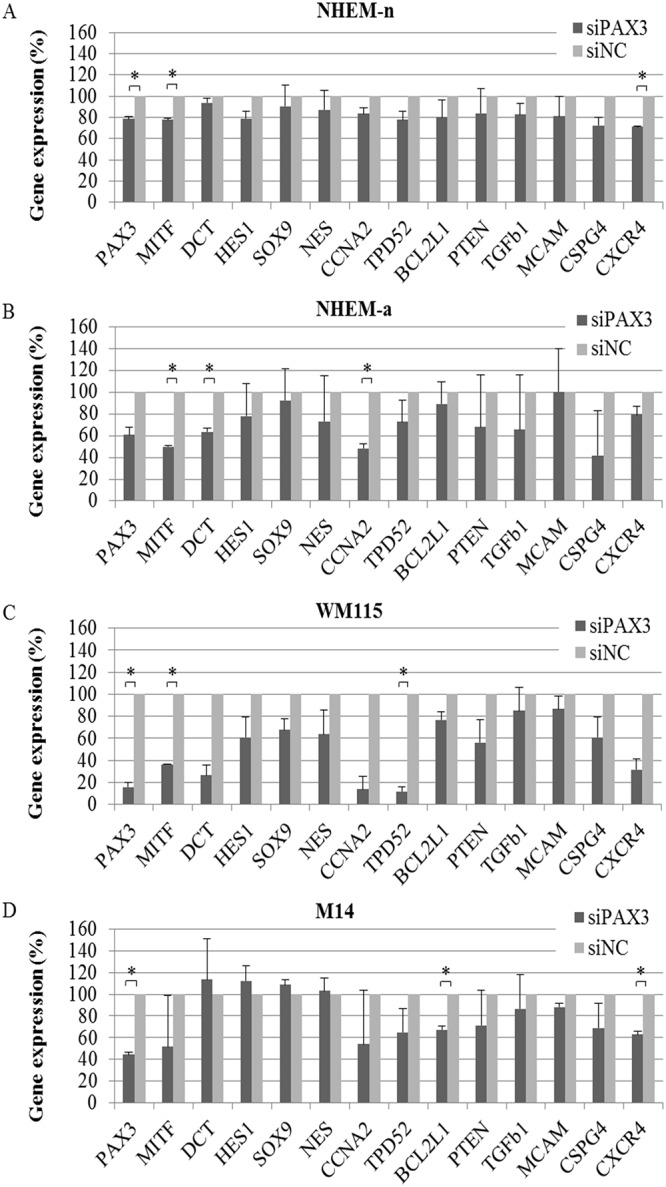
Target gene expression following *PAX3* silencing. Remaining expression levels (%) of PAX3 downstream target genes in melanocytes (NHEM-n and NHEM-a (I)) and melanoma cells (WM115 and M14) assessed two day post-transfection with *PAX3* siRNA. Cells were treated with 10 μM siPAX3#1 (s224172, Ambion) alone, with consistent results observed across several independent experiments. Expression levels of downstream target genes were normalised to 18S (ΔCt) and calculated relative to the negative control siRNA (ΔΔCt). Each silencing experiment was performed with a biological duplicate.

We first compared the efficiency of the two siRNA probes, siPAX3#1 (s224172, Ambion) and siPAX3#2 (s10059, Ambion) transfected either alone or in combination (S1 Fig), and found the most efficient and consistent reduction in *PAX3* expression of up to 52% in melanocytes and 75% in melanoma cells, two days following transfection with siPAX3#1 alone (S2 Fig, Fig 1). siPAX3#1 was used for further experiments.

*PAX3* silencing had a limited effect on expression of select target genes in melanocytes, but did significantly reduce expression of the differentiation gene *DCT* and the proliferation gene *CCNA2*. In contrast, genes associated with cell survival *BCL2L1*, cell migration *CXCR4* and proliferation *TPD52*, were significantly affected in melanoma cell lines. As expected, a well-known PAX3 target associated with differentiation, *MITF*, was commonly down-regulated following *PAX3* silencing in all cell lines, although not significantly so in M14 cells. To confirm these findings, we investigated these downstream target gene expression profiles in the metastatic melanoma cell line, A2058, following *PAX3* silencing (data not shown) and found similar results to those observed in M14 melanoma cells.

### *PAX3* transcript expression patterns in melanocytes and melanoma cells

**Fig 2:**
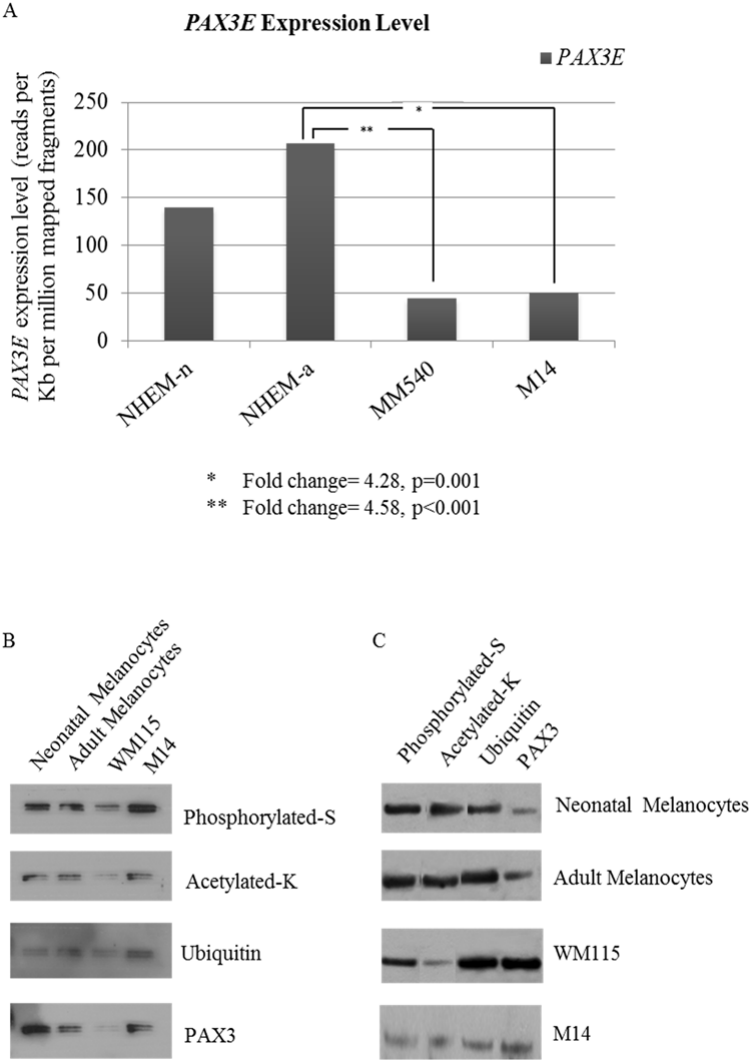
Mechanisms of differential regulation of previously identified downstream target genes of PAX3. A) A comparison of *PAX3E* expression levels between melanocytes (NHEM-n and NHEM-a) and MM540 and M14 melanoma cell lines measured as number of reads per kilobase of exon model per million mapped fragments. Asterisks (*, **) denote significant differences. B) Immunoprecipitation with Pierce Direct IP Kit, using anti-PAX3 Ab (rabbit monoclonal, Life Technologies and immunoblotting with anti-acetyl-lysine, anti-phosphoserine and anti-ubiquitin. C) Immunoprecipitation (with IgG beads) using anti-phosphoserine, anti-acetylysine, anti-ubiquitin and anti-PAX3 (rabbit polyclonal, Abcam), immunoblotted with anti-PAX3 (mouse monoclonal, DSHB) in human epidermal melanocytes (NHEM-n and NHEM-a (I)) and WM115 and M14 melanoma cell lines.

To identify the factors that may contribute to differential regulation of downstream target genes we compared *PAX3* alternate transcript expression profiles in NHEM-n and NHEM-a (P) neonatal and adult melanocytes, MM540 primary and M14 metastatic melanoma cell lines using RNAseq (Illumina HiSeq platform). One transcript, *PAX3E*, was significantly down-regulated in melanoma cells compared to melanocytes (Fig 2A), with a 4-fold reduction in *PAX3E* observed in both M14 and MM540 cells relative to adult melanocytes (NHEM-a) (p = 0.001, and p<0.001 respectively). There was no significant difference in *PAX3* transcript expression profiles between neonatal and adult melanocytes or between primary and metastatic melanoma cell lines.

### Post-translational modifications of PAX3 protein in melanocyte and melanoma cell lines

We next assessed whether the differences observed in transcript expression profiles could be due to differential post-translational modifications of the PAX3 protein in the melanocyte cultures relative to melanoma cell lines. PAX3 proteins were immunoprecipitated using anti-PAX3 antibody (rabbit monoclonal (Life Technologies), or polyclonal (Abcam)) and membranes were probed with specific antibodies that detect phosphoserine, acetyl-lysine and ubiquitin residues (Fig 2B). Additionally, immunoprecipitation was performed with anti-acetyl lysine, anti-phosphoserine and anti-ubiquitin antibodies individually, and anti-PAX3 antibody (mouse monoclonal, DSHB) was then used to probe the membranes (Fig 2C). Each precipitating antibody (anti-PAX3, anti-acetyl lysine, anti-phosphoserine and anti-ubiquitin) was run alongside its respective immunoprecipitation (IP) sample to confirm the presence of the desired product relative to the presence of antibody heavy chains (at approximately 50 kDa) (data not shown).

Our results show that the proteins corresponding to either PAX3E (56 kDa), and/or PAX3C and/or D (53 kDa), isoforms are similarly phosphorylated, acetylated and ubiquitinated in all cell lines, although acetylation occurred to a lesser degree in WM115 (Fig 2B). This was confirmed by immunopreciptation with anti-phosphoserine, anti-acetyl lysine and anti-ubiquitin antibodies followed by imunoblotting with anti-PAX3 (Fig 2C).

### PAX3 protein levels compared across multiple melanocyte cells and melanoma cell lines

**Fig 3:**
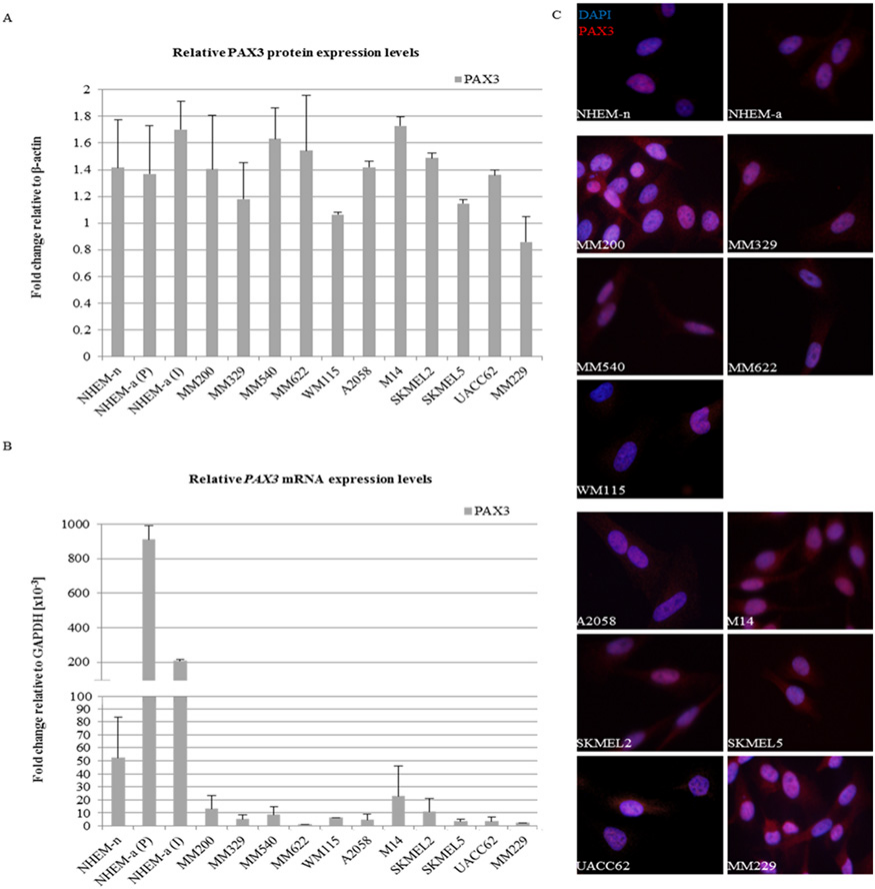
PAX3/*PAX3* expression in a representative panel of melanocytes and melanoma cell lines. A) PAX3 protein levels relative to β-actin. Each sample was run in duplicate on separate gels and the density of each band was assessed by densitometric scanning using the GS-800 Calibrated Densitometer (Bio-Rad). The density of each PAX3 band was compared to that of β-actin for each lane on the same membrane and the fold change calculated and graphed. The error bars represent the standard deviation between duplicate samples. B) *PAX3* mRNA expression in neonatal and adult normal human epidermal melanocytes and primary and metastatic melanoma cell lines. RT-qPCR was performed using RNA from neonatal (NHEM-n) and adult (NHEM-a (P) and NHEM-a (I)) human epidermal melanocytes as well as six primary (MM200, MM229, MM329, MM540, MM622 and WM115) and five metastatic (A2058, M14, SKMEL2, SKMEL5 and UACC62) melanoma cell lines. The level of *PAX3* expression was calculated as fold-change relative to *GAPDH* expression levels. All PCRs were performed in triplicate and average values were used to calculate fold change over *GAPDH* for each sample. All samples were run in biological duplicates and the error bars represent the standard deviation of the biological replicates. C) Immunocytochemistry of neonatal and adult human epidermal melanocytes, five metastatic melanoma cell lines and six primary melanoma cell lines. Nuclear PAX3 protein expression (red) is observed in all melanoma cell lines and primary melanocytes. Cell nuclei are stained with DAPI (blue). The scale measures 100 μM. A negative control was included with each experimental run and proved negative in each instance.

We next measured PAX3 protein levels in three normal melanocyte cultures (neonatal NHEM-n, and adult NHEM-a (P) and NHEM-a (I)), and eleven melanoma cell lines (primary MM200, MM229, MM329, MM540, MM622, WM115, and metastatic A2058, M14, SKMEL2, SKMEL5, UACC62 cells) by both immunocytochemistry and western blot to ensure our observed results were not cell line specific. Similar levels of PAX3 protein were observed in all melanoma cell lines and in melanocytes (p = 0.088) (Fig 3A). Using immunocytochemistry we confirmed that indeed 90–95% of cells were PAX3 positive in all cell types (Fig 3C).

All cells demonstrated the presence of the predominant band corresponding either to PAX3E (56 kDa) or to PAX3C or D isoforms (both predicted to be 53 kDa), and there was no significant difference in relative PAX3 protein levels between the melanoma cell lines and melanocytes when assessed by Western Blot (Fig 3A).

### *PAX3* mRNA levels compared across multiple melanocyte cells and melanoma cell lines

Again to confirm that our results were not cell line specific, we also compared total *PAX3* mRNA expression levels in the three melanocyte cultures (neonatal NHEM-n, and adult NHEM-a (P) and NHEM-a (I)) and the eleven melanoma cell lines (primary MM200, MM229, MM329, MM540, MM622, WM115, and metastatic A2058, M14, SKMEL2, SKMEL5, UACC62 cell lines) (Fig 3B).

A significant difference in total *PAX3* mRNA levels was observed between groups (p = 0.035), with *PAX3* mRNA significantly higher in melanocytes than in melanoma, either primary (p = 0.001) or metastatic melanoma cell lines (p = 0.002). There was no difference in *PAX3* expression levels between primary and metastatic melanoma cell lines (p = 0.644) but significantly higher levels of *PAX3* mRNA were observed in adult melanocytes, both NHEM-a (P) and NHEM-a (I), than in neonatal melanocytes (p = 0.006, p = 0.004, respectively). Furthermore, *PAX3* mRNA expression was significantly higher in adult melanocytes compared to both primary and metastatic melanoma cells (p = 0.004, p = 0.005, respectively) (Fig 3B).

### PAX3-MITF-BRN2 regulation axis in melanocytes and melanoma cells

**Fig 4:**
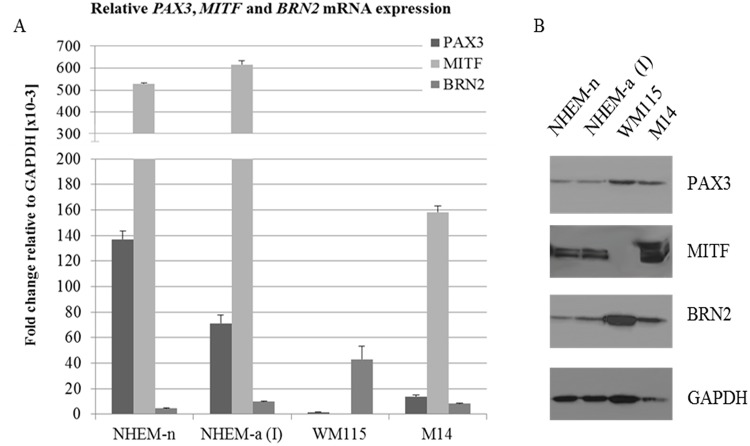
PAX3-MITF-BRN2 regulation axis in a selected panel of melanocytes and melanoma cell lines. A) *PAX3*, *MITF* and *BRN2* mRNA expression levels in melanocytes and melanoma cells. RT-qPCR was performed to assess expression levels of *PAX3*, *MITF* and *BRN2* mRNA in primary melanocytes and melanoma cell lines. All PCRs were performed in triplicate and expression values were calculated as fold change relative to *GAPDH*. Biological replicates were performed for each sample and error bars denote the standard deviation between biological replicates. B) PAX3, MITF and BRN2 protein levels in primary melanocytes and melanoma cells. Total protein from primary melanocytes and melanoma cells was separated by SDS-PAGE and immunoblotted with anti-PAX3, anti-MITF, anti-BRN2 and anti-GAPDH.

To further discern the roles of PAX3 in melanocyte and melanoma cells, we have analysed the correlation between PAX3 and MITF and BRN2. MITF and BRN2 are two transcription factors found to be mutually exclusively expressed in melanoma cells, marking highly proliferative versus highly invasive melanoma cells, respectively [32]. We have assessed the expression of PAX3, MITF and BRN2 in two melanocyte cultures (NHEM-n and NHEM-a) and two melanoma cell lines (WM115 and M14) by RT-qPCR and western blot (Fig 4). The RT-qPCR results show a positive correlation between *PAX3* and *MITF* expression, whereas *PAX3* inversely correlates with *BRN2*. This was most noticeable in WM115 melanoma cells, showing highest *BRN2* expression and lowest *PAX3* expression, and complete loss of *MITF*. Again the discrepancy is seen for PAX3 mRNA and protein levels in WM115 cells, suggesting post-translational regulatory mechanisms are in place here.

## Discussion

Previously we identified a subset of direct target genes of PAX3 [8]. Through the use of a *PAX3* knockdown experiment, we here confirm that PAX3 differentially regulates a number of these downstream targets in melanoma cells compared to normal melanocytes. In order to assess the mechanism behind this differential regulation, transcript expression profiles of *PAX3* and post-translational modifications of the PAX3 protein were analysed in melanoma cells compared to normal melanocytes. Although there were no overall differences in post-translational modifications of PAX3 in melanoma cells compared to melanocytes, our data did reveal a difference in acetylation between primary melanoma cells compared to metastatic melanoma and melanocytes and significant down-regulation of the *PAX3E* transcript in melanoma cells compared to normal melanocytes and it is possible that this down-regulation could result in the differences seen in downstream target gene regulation.

Interestingly, the function of the *PAX3E* transcript has previously been described as reducing melanocyte proliferation and increasing apoptosis [12]. Thus the decrease in *PAX3E* expression in melanoma cells may in fact facilitate tumour cell proliferation and survival. It is possible then, that its removal allows other PAX3 isoforms to bind to their specific target genes and activate their transcription, i.e. the down-regulation of *PAX3E* in melanoma cells may facilitate expression of genes responsible for proliferation and survival of melanoma cells. Similar competitive binding between isoforms has been seen for PAX5, where cell proliferation correlates with increased levels of Pax-5e whereas increased Pax-5d amounts correlate with inhibition of cell growth [33].

Post-translational modifications of PAX3 have been shown to alter the function of the PAX3 protein. PAX3 acetylation in mouse neural cells promotes neurogenesis whereas deacetylated PAX3 promotes the maintenance and proliferation of stem cells, through activation of *Neurog2* and *HES1*, respectively [16]. Furthermore, phosphorylation and ubiquitination in mouse myogenic cells, drives myoblasts to differentiate [13–15,17–19]. Our results indicate that there is no difference in phosphorylation or ubiquitination of PAX3 in melanocytes relative to melanoma cell lines; however, acetylation seems to be decreased in the primary melanoma cell line WM115 compared to the metastatic melanoma cell line and normal melanocytes. This suggests that PAX3 phosphorylation or ubiquitination are not the likely mechanisms causing differential downstream target gene regulation between melanocytes and melanoma cell lines. However, the decreased acetylation in primary melanoma compared to metastatic melanoma and melanocytes suggests that this mechanism may affect downstream target gene regulation in this cell population and warrants further investigation.

RT-qPCR analysis of *PAX3* expression in melanocytes and melanoma cells revealed significantly higher expression of the overall *PAX3* mRNA in melanocytes compared to any of the melanoma cell lines analysed. Analysis of PAX3 protein expression, however, shows no significant difference in PAX3 protein levels between any of the cell types. While post-translational modifications of the PAX3 protein cannot explain its increased degradation, inhibition of *PAX3* mRNA translation can be occurring through miRNA regulation. This warrants further investigation into the role of miRNA in regulation of PAX3 protein levels.

One miRNA that can potentially affect PAX3 protein translation is miR-211, encoded within the MITF-regulated gene melastatin (*TRPM1*) [34]. miR-211 is shown to repress the POU3F2/BRN2 transcription factor in normal melanocytes, and it is decreased in metastatic melanoma cells compared to normal melanocytes [34]. This is in concordance with the phenotype switching model, where highly invasive melanoma cells show high BRN2 expression and proliferative melanoma cells show an MITF-regulated gene expression profile [35,36]; thus BRN2 and MITF act mutually exclusively. *PAX3* mRNA also contains a single miR-211 binding site, and the over-expression of miR-211 reduces *PAX3* expression in melanoma cells (data not shown). However, this matches the observed decrease of BRN2 in these lines, and could be a result of the apparent co-regulation of both *PAX3* and *BRN2*. BRN2 can transcriptionally regulate both *PAX3* and *MITF* [37,38]; however, both PAX3 and MITF can also regulate *BRN2* expression, in a positive and negative feedback loop, respectively [34,38]. Our results confirm that in melanocytes PAX3 directly regulates *MITF* and both PAX3 and MITF expression inversely correlate with that of BRN2. The discrepancy between high mRNA and relatively low protein levels for PAX3 that we observe in melanocytes can potentially be attributed to the miR-211 interference in *PAX3* mRNA translation. By contrast, in the WM115 melanoma cell line, even though *PAX3* mRNA levels are low (compared to melanocytes), the complete lack of MITF can result in lack of translational interference though miR-211, allowing PAX3 protein translation. This is accompanied by increased BRN2 expression, due to either direct transcriptional regulation by PAX3, or lack of translational repression by miR-211, or both. The precise relationship and the interplay between PAX3, MITF and BRN2 in melanocytes and melanoma cells needs to be further investigated.

While the results presented in this paper provide further insight into the roles of PAX3 in melanocytes and melanoma cells, they may not fully explain the differential functions of PAX3. One result of note is the differential expression of alternate transcripts in melanoma cells versus melanocytes. If proven in a larger cohort, differences in *PAX3* expression profiles may, in the future, be used to stratify melanoma tumours for diagnosis, prognosis and potential treatment.

## Supporting Information

S1 Fig *PAX3* siRNA efficiency. The graphs show: A) the percentage of remaining *PAX3* expression following M14 and A2058 melanoma cell transfection with 10nM of either siPAX3#1 or siPAX3#2, or both siRNAs together; B) remaining *GAPDH* expression of following silencing with siGAPDH. Transient downregulation was gradually reduced after 5 days following transfection (A). Similar results were observed when using the control siGAPDH (B). *PAX3* and *GAPDH* expression was normalised to 18S (ΔCt) and calculated relative to the negative control siRNA transfection (ΔΔCt).C) Average *PAX3* expression in melanoma cells (M14, A2058) and melanocytes (NHEM-a, NHEM-n) following silencing with siPAX3#1. ‘n’ indicated the number of biological replicates.
(TIF)

S2 Fig *PAX3* and *GAPDH* silencing efficiency in samples used in analysis. The graphs depict the percentage of remaining *GAPDH* and *PAX3* gene expression over three days following knockdown with siGAPDH and siPAX3, respectively, in A) neonatal normal human epidermal melanocytes (NHEMn), B) adult normal human epidermal melanocytes (NHEMa), C) primary melanoma cell line WM115, D) metastatic melanoma cell line M14 and E) metastatic melanoma cell line A2058. *PAX3* and *GAPDH* expression was normalised to 18S (ΔCt) and calculated relative to the negative control siRNA transfection (ΔΔCt).
(TIF)

S1 Table List of RT-qPCR primers used for analysis of the effect of *PAX3* knockdown on previously identified downstream target genes. (TIF)
