# Supplementary figures and images for: Mechanisms Contributing to Differential Regulation of PAX3 Downstream Target Genes in Normal Human Epidermal Melanocytes versus Melanoma Cells
Source: PLoS One. 2015 Apr 16;10(4):e0124154. doi: 10.1371/journal.pone.0124154 (PMC4399949; doi:10.1371/journal.pone.0124154)

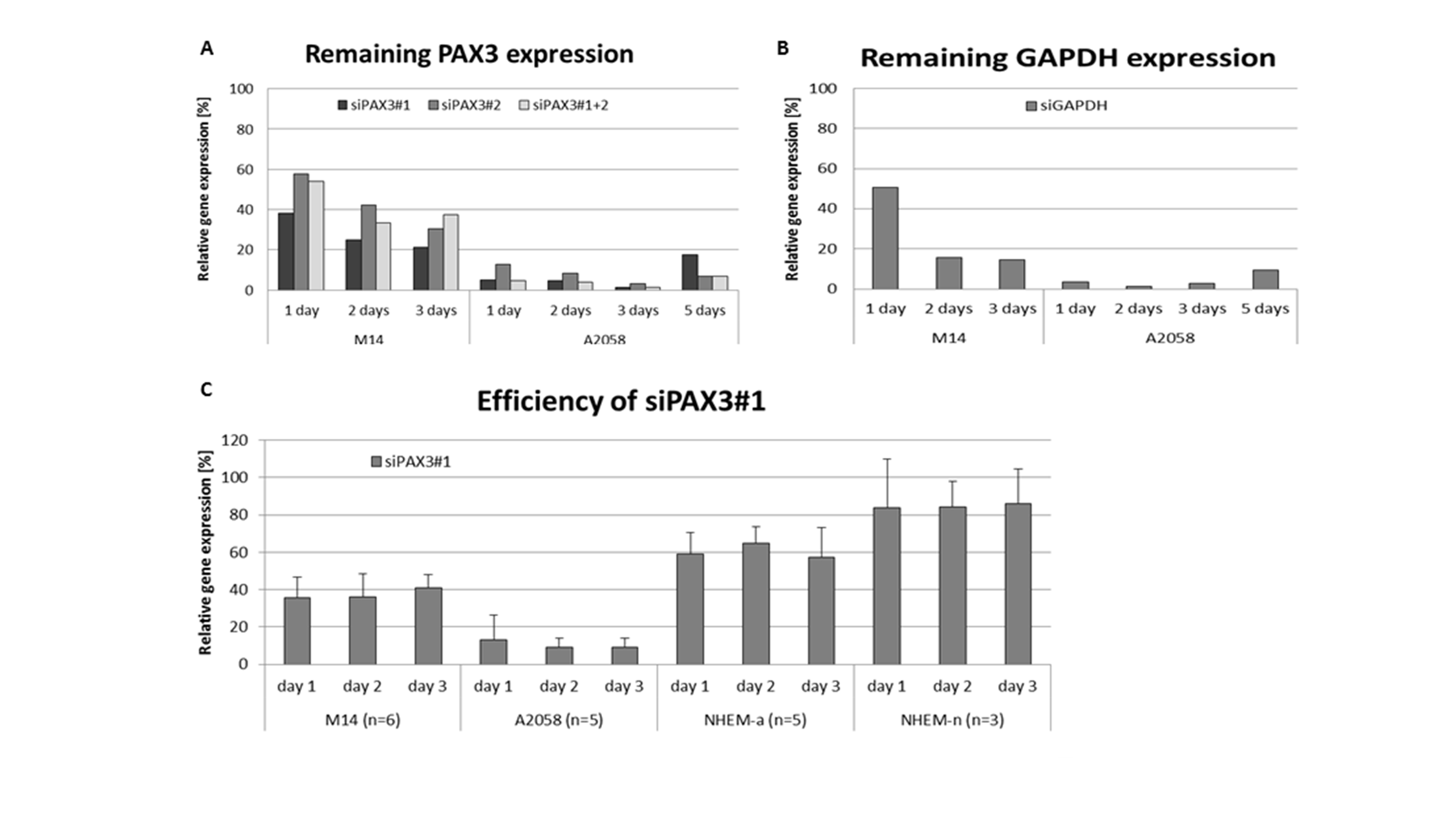

Supplement: S1 Fig — The graphs show: A) the percentage of remaining PAX3 expression following M14 and A2058 melanoma cell transfection with 10nM of either siPAX3#1 or siPAX3#2, or both siRNAs together; B) remaining GAPDH expression of following silencing with siGAPDH. Transient downregulation was gradually reduced after 5 days following transfection (A). Similar results were observed when using the control siGAPDH (B). PAX3 and GAPDH expression was normalised to 18S (ΔCt) and calculated relative to the negative control siRNA transfection (ΔΔCt).C) Average PAX3 expression in melanoma cells (M14, A2058) and melanocytes (NHEM-a, NHEM-n) following silencing with siPAX3#1. ‘n’ indicated the number of biological replicates. (TIF) [file pone.0124154.s001.tif]

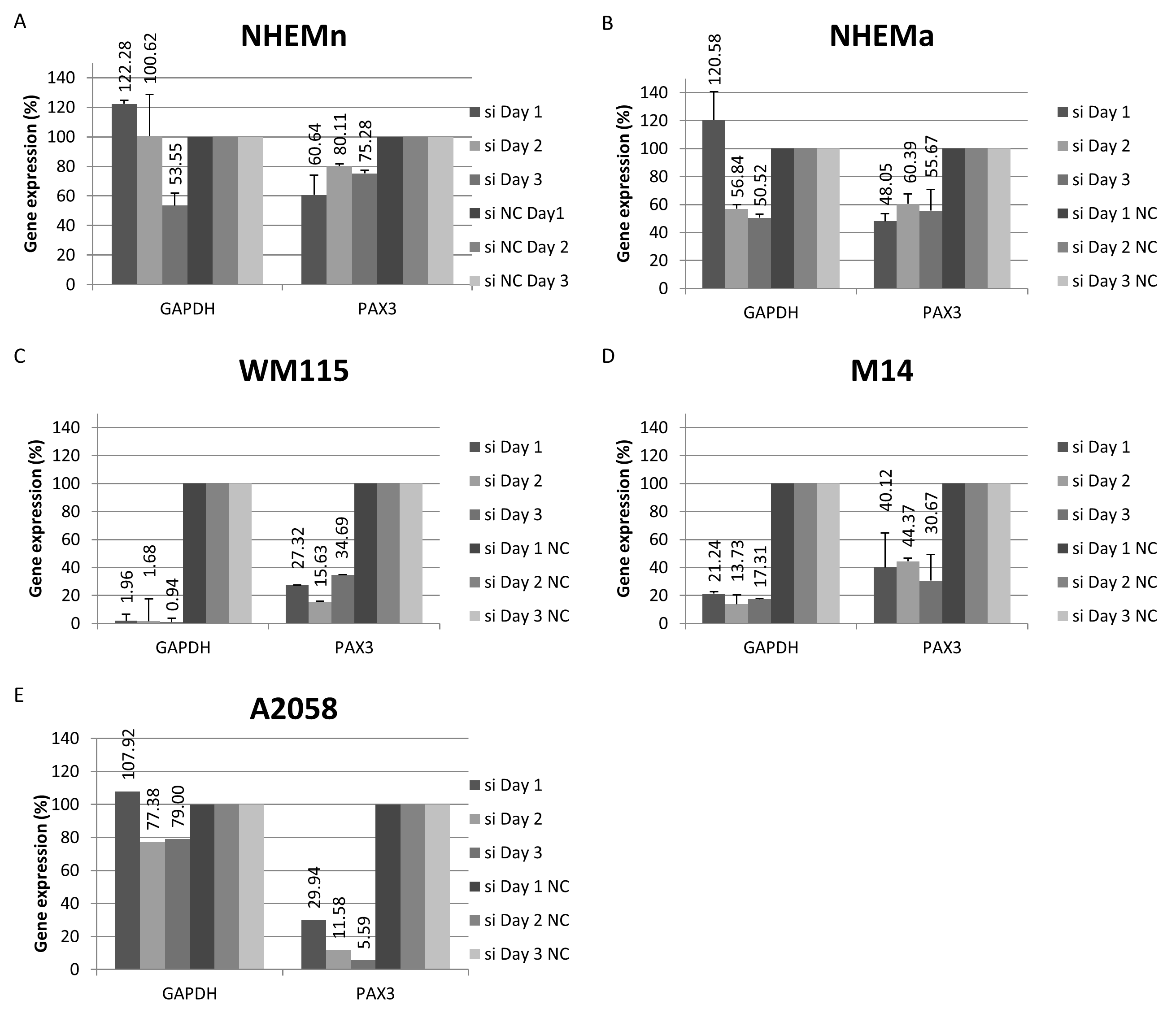

Supplement: S2 Fig — The graphs depict the percentage of remaining GAPDH and PAX3 gene expression over three days following knockdown with siGAPDH and siPAX3, respectively, in A) neonatal normal human epidermal melanocytes (NHEMn), B) adult normal human epidermal melanocytes (NHEMa), C) primary melanoma cell line WM115, D) metastatic melanoma cell line M14 and E) metastatic melanoma cell line A2058. PAX3 and GAPDH expression was normalised to 18S (ΔCt) and calculated relative to the negative control siRNA transfection (ΔΔCt). (TIF) [file pone.0124154.s002.tif]

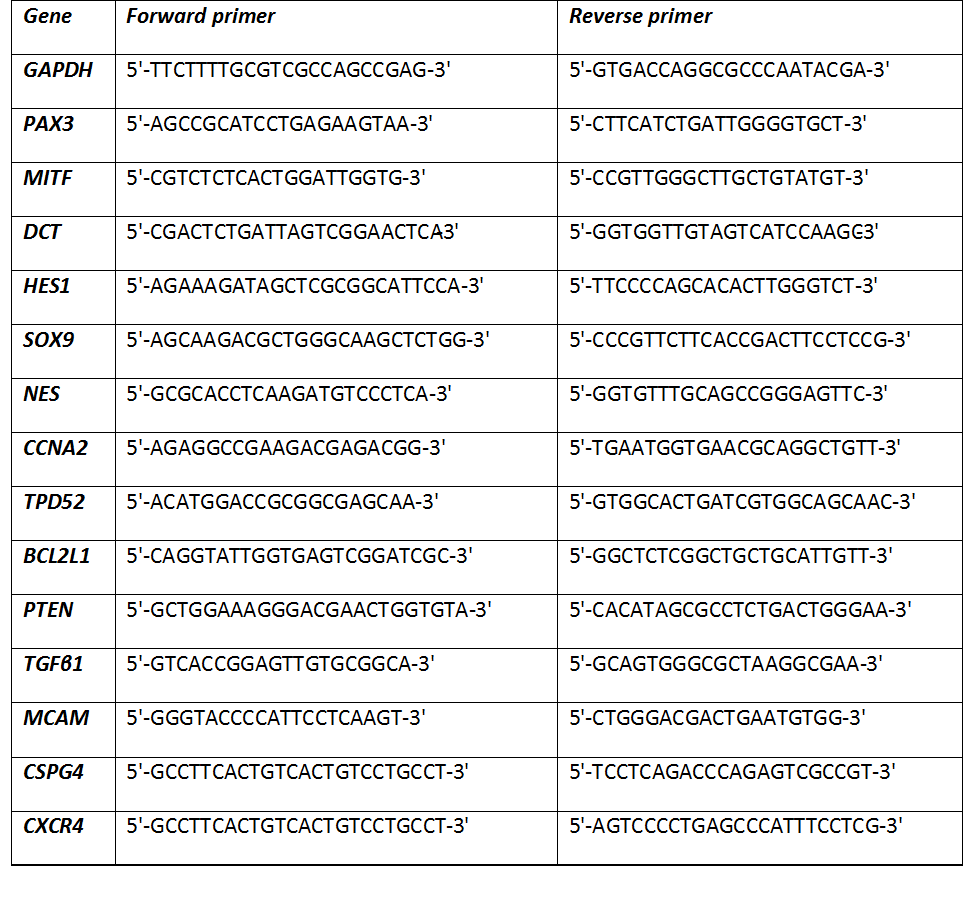

Supplement: S1 Table — (TIF) [file pone.0124154.s003.tif]
